# Multiple Beneficial Health Effects of Natural Alkylglycerols from Shark Liver Oil

**DOI:** 10.3390/md8072175

**Published:** 2010-07-19

**Authors:** Anne-Laure Deniau, Paul Mosset, Frédérique Pédrono, Romain Mitre, Damien Le Bot, Alain B. Legrand

**Affiliations:** 1 Laboratoire de Pharmacologie Moléculaire, Université de Rennes 1, France; E-Mails: anne-laure.deniau@chu-rennes.fr (A.-L.D.); frederique.pedrono@agrocampus-ouest.fr (F.P.); romain.mitre@gmail.com (R.M.); damien.lebot@univ-rennes1.fr (D.L.B.); 2 Ecole Nationale Supérieure de Chimie de Rennes and Université Européenne de Bretagne, France; E-Mail: paul.mosset@ensc-rennes.fr

**Keywords:** alkylglycerols, shark liver oil, ether lipids, immunostimulation, anti-tumor activities

## Abstract

Alkylglycerols (alkyl-Gro) are ether lipids abundant in the liver of some elasmobranch fish species such as ratfishes and some sharks. Shark liver oil from *Centrophorus squamosus* (SLO), or alkyl-Gro mix from this source, have several *in vivo* biological activities including stimulation of hematopoiesis and immunological defences, sperm quality improvement, or anti-tumor and anti-metastasis activities. Several mechanisms are suggested for these multiple activities, resulting from incorporation of alkyl-Gro into membrane phospholipids, and lipid signaling interactions. Natural alkyl-Gro mix from SLO contains several alkyl-Gro, varying by chain length and unsaturation. Six prominent constituents of natural alkyl-Gro mix, namely 12:0, 14:0, 16:0, 18:0, 16:1 n-7, and 18:1 n-9 alkyl-Gro, were synthesized and tested for anti-tumor and anti-metastatic activities on a model of grafted tumor in mice (3LL cells). 16:1 and 18:1 alkyl-Gro showed strong activity in reducing lung metastasis number, while saturated alkyl- Gro had weaker (16:0) or no (12:0, 14:0, 18:0) effect. Multiple compounds and mechanisms are probably involved in the multiple activities of natural alkyl-Gro.

## 1. Multiple Biological Activities of Alkylglycerols

Natural 1-*O*-alkylglycerols (alkyl-Gro) are bioactive ether lipids present in body cells and fluids. They are precursors of ether phospholipids, which participate in structures and functions of membranes in certain cells such as white blood cells or macrophages. Alkyl-Gro are also found in bone marrow lipids and in milk [1].

Marine sources of alkyl-Gro such as the liver oil of certain shark species or rat fish (elasmobranch fishes) contain high levels of these compounds as a mixture of few species varying by length and unsaturation of the alkyl chain [2].

The usual composition of alkyl chains in alkyl-Gro from Greenland shark (*Centrophorus squamosus*) liver oil (SLO) is as follow: 12:0, 1–2%. 14:0, 1–3%; 16:0, 9–13%; 16:1n-7, 11–13%; 18:0, 1–5%; 18:1n-9, 54–68%; 18:1n-7, 4–6%, and minor species (<1%).

Beneficial effects of SLO on health have been recognized in traditional medicine of northern countries involved in fishing, such as Japan, Norway or Iceland. In these countries, the ancestral use of SLO was empirical as strengthening or wound healing medication.

Experimental studies were performed during the last century, aiming to demonstrate whether alkyl-Gro from SLO had biological properties and beneficial effects. Indeed, several studies did observe interesting effects, such as hematopoiesis stimulation [3], lowering radiotherapy-induced injuries [4], reducing tumor growth [5], or improving vaccination efficiency [6,7]. However, in most cases, conclusions were mainly impaired by the poor definition of the mixtures used, in terms of purity as well as chemical composition. Furthermore, none of these ancient publications studied or even suggested mechanisms for alkyl-Gro multiple effects.

For several decades, the family of bioactive lipids has grown tremendously, and lipids are not anymore seen only as fat and enemy of good health. All cells possess lipids and phospholipids, which are essential components of membranes, and membrane lipids are also used as precursors for mediators and messengers. Lipids and phospholipids have prominent roles in cell-to-cell communication and in intracellular signals as well. Many lipid-derived bioactive molecules are agonists of specific membrane receptors: besides prostanoids and leukotriens, recent discoveries of new lipid mediators include docosanoids and many other eicosanoids such as isoprostanes, anandamide, 2-arachidonoylglycerol and other natural lipids acting as cannabinoid receptor agonists. Phospholipidic mediators are as well numerous, ubiquitous and of first rank interest; they include Platelet-activating Factor (PAF), lysophosphatidic acid (Lyso-PA), lysophosphatidylcholine (Lyso-PC), and shingosine-1-P (SP-1P). Several lipids act as intracellular second messengers (diacylglycerols, free fatty acids) or as regulators of transcription factors (free fatty acids, Lyso-PA).

Thus, many of the signaling pathways involved in cellular functions and communications are dependent on lipids. Where do cell membrane lipids come from? Interestingly, lipids are the single biochemical class that allows variability in structure depending on the nutrition. Therefore, one may modify, by nutritional supply, the lipid composition of cell membranes and as a result, the structures and functions of membrane–derived bioactive lipids.

The main hypothesis for explaining multiple biological activities of alkyl-Gro was that they may be incorporated into cell membrane phospholipids, and from there modify their physical properties such as membrane fluidity and antioxidant status [8], or alter cell signaling through the phospholipase pathways. This concept has already been used in the multiple applications of n-3 fatty acid food supplementation. Its extension to alkyl-Gro is clearly a help for understanding their multiple biological activities.

## 2. Incorporation into Mediators and Second Messengers

Indeed, alkyl-Gro can be incorporated into the phospholipids of several cell types such as THP1 monocytes [9], endothelial cells [10] or blood platelets [11]. In endothelial cells, resulting 1-*O*-alkylglycerophospholipids participated in the production of 1-*O*-alkyl-2-acylglycerol [10], an analog of diacylglycerol (DAG) with inhibiting effect on protein kinase C [12]. In transformed human monocyte THP1 cells, alkyl-Gro were incorporated into 1-*O*-alkylglycerophosphatidylcholine, the precursor of Platelet-activating Factor (PAF) [9], and incorporation of [^3^H] alkyl-Gro resulted in the production of labeled PAF. Furthermore, alkyl-Gro incorporation resulted in amplifying and extending the PAF production level after stimulation [9]. Increasing 1-*O*-alkyl-2-acyl-glycerophosphocholine in membrane phospholipids could also increase the production of active PAF analogs by oxidation of unsaturated fatty acid in the 2-position of glycerol [13,14].

## 3. Male Fertility

Spermatozoa produce PAF, and this mediator plays an important role in sperm physiology with respect to capacitation, acrosome reaction and gamete fusion [15,16]. An attractive hypothesis was that alkyl-Gro, which are PAF precursors, might have effects on sperm functions. Indeed, boar sperm treated *in vitro* with a mixture of natural alkyl-Gro had increased percent motility and velocity parameters. Furthermore, such treated sperm had better fertilization performances when used for artificial inseminations [17], and this has found applications in breeding of several mammalian species. *In vivo* studies in boars showed that oral intake of SLO (40 g/day, 28 days) improved sperm motility and velocity, together with an increase in the levels of alkyl-Gro in sperm [18]. The possibility of improving fertility by oral SLO supplementation is suggested by these data, however, needs confirmation.

## 4. Immuno-Stimulation and Hematopoiesis

Since, on the one hand, ancient studies had shown that some alkyl-Gro stimulate hematopoiesis [3] and antibody production [6], and, on the other hand, alkyl-Gro are found in body fluids including milk [1], we hypothesized that oral treatment of pregnant animals could have beneficial effects on offspring health. Indeed, oral treatment by SLO in pregnant sows resulted in the amplification of vaccination-induced raise in specific immunoglobulins in serum of treated sows. This amplification of specific antibodies was also observed in colostrum of treated sows, and as expected, in serum of piglets from treated mothers. The piglets from treated sows had also higher leukocyte levels by raising populations of polymorphonuclear cells, lymphocytes and monocytes [19]. This resulted in an overall improvement in offspring health status and growth, however, besides alkyl-Gro activities, some of these beneficial effects might also result from the presence of n-3 polyunsaturated fatty acids (PUFA) in SLO [19]. In healthy humans, high doses of SLO (15 g/day) for four weeks induced changes in the cytokine profile and antioxidant status of serum together with an increase in total cholesterol and a decrease of its HDL fraction. Again, since SLO contained also high proportions of squalene and PUFA, one cannot attribute these different effects to alkyl-Gro only [20].

## 5. Anti-Tumor Activities

Using a model of solid tumor grafted in mice (Lewis lung carcinoma cells = LLC), we have evaluated the anti-tumor effects of oral SLO and of natural alkyl-Gro purified from the same source. We found that both treatments reduced significantly the growth of grafted tumors [21]. Furthermore, both treatments also reduced the number of pulmonary metastases. These data demonstrated that alkyl-Gro are active molecules accounting for anti-tumor activities of SLO. The mechanisms by which alkyl-Gro exert these anti-tumor properties may be multiple. To evaluate anti-neoangiogenic activity, we measured the density of an endothelial marker in solid tumors grafted in mice. After an alkyl-Gro oral treatment as short as five days, we established that the density of von Willebrand factor in grafted tumors was decreased by 26% as compared to control group [21]. Anti-neoangiogenic activities might be one of the possible mechanisms for anti-tumor activities of alkyl-Gro [22]. Since basic Fibroblast Growth Factor (bFGF), is a major angiogenesis stimulator [23], we studied the effect of natural mix of alkyl-Gro on endothelial cell proliferation stimulated by bFGF and showed that alkyl-Gro reduced the bFGF stimulating effect [24].

## 6. Chemical Synthesis of 1-*O*-Alkyl-*sn*-glycerols

**Figure 1:**
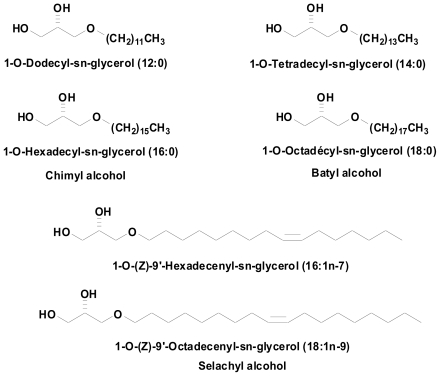
1-*O*-alkyl-sn-glycerols obtained by chemical synthesis.

Since most studies describing alkyl-Gro effects were obtained using a mix of several natural compounds, it was of interest to explore the activity of each single compound. We synthesized six of the major of natural alkyl-Gro mix, namely: (1) AKG 12:0 = 1-*O*-Dodecyl-*sn*-glycerol, (2) AKG 14:0 = 1-*O*-Tetradecyl-*sn*-glycerol, (3) AKG 16:0 = 1-*O*-Hexadecyl-*sn*-glycerol (chimyl alcohol), (4) AKG 18:0 = 1-*O*-Octadecyl-*sn*-glycérol (batyl alcohol), (5) AKG 16:1 = 1-*O-(Z)*-9′-Hexadecenyl*sn*- glycerol, (6) AKG 18:1 = 1-*O-(Z)*-9′-Octadecenyl-*sn*-glycerol (selachyl alcohol) (Figure 1).

Saturated and monounsaturated (*S*)-1-*O*-alkylglycerols were synthesized in two and four steps, respectively, from (*R*)-(−)-2,2-dimethyl-1,3-dioxolane-4-methanol. Alkylation of this latter compound by 1-bromohexadecane and 1-bromooctadecane under basic conditions (KOH, *n*-Bu_4_NBr cat., DMSO, 60 °C) followed by acetonide cleavage under acid conditions (0.05 equivalent of *p*-toluenesulfonic acid monohydrate in MeOH:H_2_O 10:1) afforded chimyl and batyl alcohols, which were purified by recrystallization in petroleum ether and methanol, respectively. Selachyl alcohol and its lower homolog, (*S*)-3-[(9*Z*)-9-hexadecen-1-yloxy]-1,2-propanediol, were respectively synthesized from oleic and palmitoleic acids. These monounsaturated fatty acids were reduced by Red-Al to oleyl and palmitoleyl alcohols. Their mesylation under classic conditions afforded the corresponding mesylates. Alkylation of (*R*)-(−)-2,2-dimethyl-1,3-dioxolane-4-methanol by these mesylates afforded the protected targets as acetonides which under the same acid conditions as previously afforded selachyl alcohol and (*S*)-3-[(9*Z*)-9-hexadecen-1-yloxy]-1,2-propanediol.

## 7. Which 1-*O*-Alkylglycerols Have Anti-Tumor Activities?

The activities of alkyl-Gro natural mixture and of each of these compounds were compared using the same *in vivo* model of solid tumor grafted in mice, using olive oil treatment as a control [24]. We found that AKG 16:1 and 18:1 were the most potent compounds on tumor growth and lung macrometastasis number, while other compounds (AKG 16:0 and 12:0) had weaker activities. We also observed that AKG 18:0 did not reduce, but on the contrary, tended to increase tumor growth and metastasis number [25].

We also observed important variations in spleen weights at the end of the experiment (day 20). In non-grafted mice, natural mix of alkyl-Gro had no effect on spleen weights. Tumor graft induced a strong increase in spleen weights of the control group. Alkyl-Gro treatments resulted in a reduction of spleen weights in most groups, with stronger effects observed in AKG 16:1 and 18:1 groups, in which spleen weights decreased to levels similar of those in non-grafted groups. Again, and by contrast with the other compounds, spleen weight in AKG 18:0-treated group was significantly increased as compared with olive oil-treated group [25].

## 8. Cytotoxicity and Effects of Alkylglycerols on Endothelial Cell Proliferation

On human umbilical vein endothelial cells (HUVEC), MTT test of cytotoxicity [26] revealed absence of cytotoxic effects after 72 hours at concentrations lower than 20 μM for any synthetic alkyl-Gro. At concentrations devoid of cytotoxic effects, synthetic 18:1 and 16:1 alkyl-Gro again had the strongest effects on [^3^H]-thymidine incorporation into HUVEC, indicating that these two unsaturated compounds might have the strongest inhibiting effect on neo-angiogenesis involved in tumor development [25].

## 9. Alkylglycerols and the Blood-Brain Barrier

Synthetic short chain alkyl-Gro such as 1-*O*-pentyl *sn* glycerol are interesting compounds, which transiently open the blood brain barrier (BBB), allowing increased crossing of the BBB by therapeutic molecules [27,28]. Parenteral 1-*O*-decyl-*sn*-glycerol may also increase carbamazepine crossing through BBB [29]. Oxidation of the natural unsaturated alkyl-Gro on the double bound could lead to short-chain alkyl-Gro with putative similar activities.

## 10. Concluding Remarks

Several biochemical and physiological mechanisms may be involved in the multiple effects of alkyl-Gro. In spermatozoa, the stimulating and protecting effects of natural alkyl-Gro were partially reduced in the presence of a PAF-receptor antagonist, indicating that interaction of alkyl-Gro with PAF metabolism and/or activities [17]. In blood platelets, alkyl-Gro partially inhibited the PAF-induced release of serotonin *in vitro*, suggesting that natural alkyl-Gro might act as a partial agonist (agonist-antagonist) on the PAF receptor [11].

For a long time, it has been established that injection of batyl alcohol in rats and guinea pigs increases the number of erythrocytes, leukocytes or platelets in blood [3,30]; by contrast selachyl alcohol—the mono-unsaturated analog of batyl alcohol—is devoid of such activities [30,31], while this compound showed strong anti-tumor activities.

Since alkyl-Gro are found in hematopoietic organs such as bone marrow, one may hypothesize that they have an important role in hematopoiesis, and could behave as precursors of PAF, which has stimulating functions in bone marrow cell lines [32,33]. Active PAF-analog production could also result from oxidation and cleavage of poly-unsaturated fatty acids at the *sn*-2 position of the 1-*O*-alkylphospholipids [13,14].

In pigs, oral SLO increased both hematopoiesis and immunoglobulin production. These effects could also be part of the mechanisms involved in anti-tumor effects of alkyl-Gro by raising immuno-competent cell populations. Furthermore, alkyl-Gro have been shown to activate macrophages *in vivo* [34], but only in the presence of non-adherent B an T cells *in vitro*, suggesting multi-factorial cell-to-cell interactions [35]. Direct stimulating activity of alkyl-Gro on calcium signaling in human Jurkat T lymphocytes has also been observed *in vitro* [36].

Among the synthetic compounds tested, the anti-tumor and anti-metastasis activities of natural alkyl-Gro from SLO are mostly supported by the unsaturated compounds, which have 16 and 18 carbon alkyl chains. These activities are associated with a strong reduction of tumor-induced increase in spleen weight. These two compounds represent mainly 70 to 80% of total alkyl-Gro from SLO, explaining activity of natural mix of alkyl-Gro. However, the natural mix contains a minor compound, AKG 18:0 which has remarkable opposite effects on spleen weight. Therefore the use of the two most active compounds could be of interest for improving anti-tumor and anti-metastasis efficiency of alkyl- Gro. The splenomegaly observed after 3LL cell graft reflects spleen invasion by immature myeloid cells, predominantly neutrophil granulocytes, and is associated with a decrease in blood B and T lymphocytes [37,38], both events impairing efficient immune responses to tumor cells. Amplification of this response by the hematopoietic effect of batyl alcohol might explain spleen size rise induced by this compound together with its inactivity on tumor growth and metastasis. The mechanism of spleen size reduction observed in grafted mice treated with AKG 18:1 and 16:1 is poorly understood; it could however contribute to restore immune defenses against tumor cells. Activities of products resulting from double bound oxidation could be part of the difference between unsaturated *versus* saturated alkyl-Gro anti-tumor activities.

Mechanisms of anti-tumor effects could also be related to anti-angiogenic activities, since both unsaturated alkyl-Gro reduced endothelial proliferation, which is involved in neo-angiogenesis.

Several biochemical mechanisms could be involved in the multiple activities of alkyl-Gro. Their role in increasing PAF precursor and amplifying PAF production [9] could explain effects related to PAF activities. These activities include inflammation and sperm functions, but also immune-regulations [39,40]. Another interesting track is the interaction of 1-*O*-alkyl-2-acylglycerol with protein kinase C. This compound, which is produced following cell stimulation after alkyl-Gro incorporation in membrane phospholipids [10], is an analog of DAG which inhibits PKC [12]; 1-*O*-alkyl-2-acylglycerol binds to, but do not activates PKC-ɛ and competes with DAG [41]; this could result in cell growth arrest as observed in endothelial cells [21]. Although the relation between unsaturation and anti-tumor activities of alkyl-Gro remains poorly understood, it appears that each molecule may have specific mechanism of action and particular activities.

**Patents**: WO 2006/024742, USA 11/453/309, FR n°0953443.

## References

[1] Hallgren B Larsson SO The glyceryl ethers in man and cow J. Lipid Res 1962 3 39 43

[2] Bordier CG Sellier N Foucault AP Le Goffic F Purification and characterization of deep sea shark Centrophorus squamosus liver oil 1-*O*-alkylglycerol ether lipids Lipids 1996 31 521 528 8727645 10.1007/BF02522646

[3] Linman JW Long MJ Korst DR Bethell FH Studies on the stimulation of hemopoiesis by batyl alcohol J. Lab. Clin. Med 1959 54 335 343 14417292

[4] Brohult A Brohult J Brohult S Joelsson I Effect of alkoxyglycerols on the frequency of injuries following radiation therapy for carcinoma of the uterine cervix Acta Obst. Gynecol. Scand 1977 56 441 448 602713 10.3109/00016347709155008

[5] Brohult A Brohult J Brohult S Regression of tumour growth after administration of alkoxyglycerols Acta Obstet. Gynecol. Scand 1978 57 79 83 622894 10.3109/00016347809154203

[6] Ngwenya BZ Foster DM Enhancement of antibody production by lysophosphatidylcholine and alkylglycerol Proc. Soc. Exp. Biol. Med 1991 196 69 75 1984244 10.3181/00379727-196-43165

[7] Brohult A Brohult J Brohult S Effect of irradiation and alkoxyglycerol treatment on the formation of antibodies after salmonella vaccination Experientia 1972 28 954 955 5076333 10.1007/BF01924969

[8] Debouzy J-C Crouzier D Lefebvre B Dabouis V Study of alkylglycerol containing shark liver oil: a physic chemical support for biological effects Drug Target Insights 2008 3 125 135

[9] Hichami A Duroudier V Leblais V Vernhet L Le Goffic F Ninio E Legrand A Modulation of platelet-activating-factor production by incorporation of naturally occurring 1-*O-*alkylglycerols in phospholipids of human leukemic monocyte-like cells THP-1 Eur. J. Biochem 1997 250 242 248 9428670 10.1111/j.1432-1033.1997.0242a.x

[10] Marigny K Pédrono F Martin-Chouly C Youmine H Saïag B Legrand AB Modulation of endothelial permeability by 1-*O*-alkylglycerols Acta Physiol. Scand 2002 176 263 268 12444931 10.1046/j.1365-201X.2002.01037.x

[11] Pédrono F Cheminade C Legrand AB Natural 1-*O*-alkylglycerols reduce platelet-activating factor-induced release of [^3^H]-serotonin in rabbit platelets Prostag. Leukot. Ess. Fatty Acids 2004 71 19 23 10.1016/j.plefa.2003.12.003 15172680

[12] Heymans F Da Silva C Marrec N Godfroid J-J Castagna M Alkyl analogs of diacylglycerols as activators of protein kinase C FEBS Lett 1987 218 35 40 3595862 10.1016/0014-5793(87)81013-x

[13] Kamido H Eguchi H Ikeda H Imaizumi T Yamana K Hartvigsen K Ravandi A Kuksis A Core aldehydes of alkyl glycerophosphocholines in atheroma induce platelet aggregation and inhibit endothelium-dependent arterial relaxation J. Lipid Res 2002 43 158 166 11792735

[14] Hartvigsen K Ravandi A Harkewicz R Kamido H Bukhave K Holmer G Kuksis A A-*O*- alkyl-2-(ω-oxo)acyl-*sn-*glycerols from shark liver oil and human milk fat are potential precursors of PAF mimics and GHB Lipids 2006 41 679 693 17069352 10.1007/s11745-006-5019-4

[15] Kraus CS Gervasi G Fori G Baldi E Effect of platelet-activating factor on motility and acrosome reaction on human spermatozoa Hum. Reprod 1994 9 471 476 8006136 10.1093/oxfordjournals.humrep.a138529

[16] Fukuda A Roudebush WE Thatcher SS Platelet-activating factor enhances the acrosome reaction, fertilization *in vitro* by subzonal sperm injection and resulting embryonic development in the rabbit Hum. Reprod 1994 9 94 99 8195361 10.1093/oxfordjournals.humrep.a138330

[17] Cheminade C Gautier V Hichami A Allaume P Le Lannou D Legrand AB 1-*O-*alkylglycerols improve boar sperm motility and fertility Biol. Reprod 2002 66 421 428 11804958 10.1095/biolreprod66.2.421

[18] Mitre R Cheminade C Allaume P Legrand P Legrand AB Oral intake of shark liver oil modifies lipid composition and improves motility and velocity of boar sperm Theriogenology 2004 62 1557 1566 15451263 10.1016/j.theriogenology.2004.02.004

[19] Mitre R Etienne M Martinais S Salmon H Allaume P Legrand P Legrand AB Humoral defence improvement and haematopoiesis stimulation in sows and offspring by oral supply of shark liver oil to mothers during gestation and lactation Br. J. Nutr 2005 94 753 762 16277779 10.1079/bjn20051569

[20] Lewkowicz P Banasik M Glowacka E Lewkowicz N Tchorzewski H Effect of high doses of shark liver oil supplementation on T cell polarization and peripheral blood polymorphonuclear cell function Pol. Merk. Lek 2005 18 686 692 16124384

[21] Pedrono F Martin B Leduc C Le Lan J Saïag B Legrand P Moulinoux JP Legrand AB Natural alkylglycerols restrain growth and metastasis of grafted tumour in mice Nutr. Cancer 2004 48 64 69 15203379 10.1207/s15327914nc4801_9

[22] Skopinska-Rózewska E Krotkiewski M Sommer E Rogala E Filewska M Bialas-Chromiec B Pastewka K Skurzak H Inhibitory effect of shark liver oil on cutaneous angiogenesis induced in Balb/c mice by syngeneic sarcoma L-1, human urinary bladder and human kidney tumour cells Oncol. Rep 1999 6 1341 1343 10523708 10.3892/or.6.6.1341

[23] Presta M Dell’Era P Mitola S Moroni E Ronca R Rusnati M Fibroblast growth factor/Fibroblast growth factor receptor system in angiogenesis Cytokine Growth Factor Rev 2005 16 159 178 15863032 10.1016/j.cytogfr.2005.01.004

[24] Pédrono F Saïag B Moulinoux J-P Legrand AB 1-*O*-alkylglycerols reduce the stimulating effects of bFGF on endothelial cell proliferation *in vitro* Cancer Lett 2007 251 317 322 17207571 10.1016/j.canlet.2006.11.028

[25] Deniau A-L Mosset P Le Bot D Legrand AB Which alkylglycerols from shark liver oil have anti-tumour activities? Biochimie 2010 10.1016/j.biochi.2009.12.010 20036307

[26] Mosmann T Rapid colorimetric assay for cellular growth and survival: application to proliferation and cytotoxicity assays J. Immunol. Meth 1983 65 55 63 10.1016/0022-1759(83)90303-4 6606682

[27] Erdlenbruch B Jendrossek V Eibl H Lakomek M Transient and controllable opening of the blood-brain barrier to cytostatic and antibiotic agents by alkylglycerols in rats Exp. Brain Res 2000 135 417 422 11146820 10.1007/s002210000553

[28] Erdlenbruch B Jendrossek V Kugler W Eibl H Lakomek M Increased delivery of erucylphosphocholine to C6 gliomas by chemical opening of the blood-brain barrier using intracarotid alkylglycerols in rats Cancer Chemother. Pharmacol 2002 50 299 304 12357304 10.1007/s00280-002-0497-4

[29] Madhusudhan B Rambhau D Apte SS Gopinath D 1-O-alkylglycerol stabilized carbamazepine intraveinous o/w nanoemulsions for drug targeting in mice J. Drug Targeting 2007 15 154 161 10.1080/10611860601141150 17365287

[30] Osmond DG Roylance PJ Webb AJ Yoffey JM The action of batyl alcohol and selachyl alcohol on the bone marrow of guinea pig Acta Haemat 1963 29 180 186 13940548 10.1159/000207341

[31] Linman JW Hematopoietic effects of glyceryl ethers. III. Inactivity of selachyl alcohol Proc. Soc. Exp. Biol. Med 1960 104 703 706 13762473 10.3181/00379727-104-25959

[32] Denizot Y Guglielmi L Donnard M Trimoreau F Platelet-activating factor and normal and leukaemic haematopoiesis Leuk. Lymphoma 2003 44 775 782 12802913 10.1080/1042819031000067549

[33] Dupuis F Levasseur S Jean-Louis F Dulery C Praloran V Denizot Y Michel L Production, metabolism and effect of platelet-activating factor on the growth of the human K562 cell line Bichim. Biophys. Acta 1997 1359 241 249 10.1016/s0167-4889(97)00106-7 9434130

[34] Yamamoto N Ngwenya BZ Sery TW Pieringer RA Activation of macrophages by ether analogues of lysophospholipids Cancer Immunol. Immunother 1987 25 185 192 2824051 10.1007/BF00199146 PMC11038200

[35] Yamamoto N St Claire DA Jr Homma S Ngwenya BZ Activation of mouse macrophages by alkylglycerols, inflammation products of cancerous tissues Cancer Res 1988 48 6044 6049 3167855

[36] Pédrono F Khan NA Legrand AB Regulation of calcium signalling by 1-O-alkylglycerols in human Jurkat T cells Life Sci 2004 74 2793 2801 15043993 10.1016/j.lfs.2003.11.002

[37] Lee J-K Back TC Komschlies KL Ruscetti FW Young HA Wiltrout RH Hematopoietic switch from lymphoid to granulocytic development in 3LL tumor-bearing mice In Vivo 2001 15 255 264 11695215

[38] Shin-Ya M Mazda O Tsuchihara C Hirai H Imanishi J Takeuchi M Interleukin-2 abolishes myeloid cell accumulation induced by Lewis lung carcinoma J. Interferon Cytokine Res 2003 23 631 638 14651777 10.1089/107999003322558764

[39] Al-Darmaki S Knightshead K Ishihara Y Best A Schenkein HA Tew JG Barbour SE Delineation of the role of platelet-activating factor in the immunoglobulin G2 antibody response Clin. Diagn. Lab. Immunol 2004 4 720 728 15242947 10.1128/CDLI.11.4.720-728.2004 PMC440608

[40] Matsumura Y Byrne SN Nghiem DX Miyahara Y Ullrich SE A role for inflammatory mediators in the induction of immunoregulatory B cells J. Immunol 2006 177 4810 4817 16982922 10.4049/jimmunol.177.7.4810 PMC1579249

[41] Houck KL Fox TE Sandirasegarane L Kester M Ether-linked diglycerides inhibit vascular smooth muscle cell growth via decreased MAPK and PI3K/Akt signalling Am. J. Physiol 2008 295 1657 1668 10.1152/ajpheart.00141.2008 PMC2593499 18723771

